# Role and mechanism of Twist1 in modulating the chemosensitivity of FaDu cells

**DOI:** 10.3892/mmr.2014.2212

**Published:** 2014-05-06

**Authors:** SUMEI LU, LIANG YU, YAKUI MU, JUKE MA, JIAJUN TIAN, WEI XU, HAIBO WANG

**Affiliations:** 1 Department of Otorhinolaryngology Head and Neck Surgery, Shandong Provincial Hospital Affiliated to Shandong University, Jinan, Shandong 250022, P.R. China; 2 Department of Laboratory Medicine, Shandong Provincial Qianfoshan Hospital, Shandong University, Jinan, Shandong 250014, P.R. China; 3 Shandong Provincial Key Laboratory of Otology, Shandong Provincial Hospital Affiliated to Shandong University, Jinan, Shandong 250022, P.R. China; 4 Institute of Eye and ENT, Shandong Provincial Hospital Affiliated to Shandong University, Jinan, Shandong 250022, P.R. China

**Keywords:** Twist1, multidrug resistance, head and neck squamous cell carcinoma, chemosensitivity, cell apoptosis

## Abstract

Multidrug resistance (MDR) is one of the most important obstacles affecting the efficacy of chemotherapy treatments for numerous types of cancer. In the present study, we have demonstrated the possible function of Twist1 in the chemosensitivity of head and neck squamous cell carcinoma (HNSCC) and have identified that its mechanism maybe associated with MDR1/P-gp regulation. To investigate this, the hypopharyngeal cancer cell line, FaDu, and its MDR cell line induced by taxol, FaDu/T, were employed. Stable transfectants targeted to Twist1 overexpression and Twist1 silencing based on FaDu were also conducted. Morphological observation, flow cytometry, reverse transcription-polymerase chain reaction (RT-PCR), western blotting and laser scanning confocal microscope detection were utilized to detect the associations between Twist1 and the chemosensitivity of FaDu cells. Our results demonstrated that Twist1 and MDR1/P-gp were upregulated in FaDu/T cells in a MDR dose-dependent manner. The anti-apoptotic capabilities of FaDu/T cells were enhanced during MDR progression, with apoptosis-related proteins (Bcl-2, Bax, activated caspase-3 and caspase-9) changing to resist apoptosis. Twist1 overexpression decreased the sensitivity of cells to taxol as revealed by a significant increase in MDR1/P-gp and IC_50_ (P<0.05). This overexpression also enhanced the resistance to apoptosis, with apoptotic proteins changing to resist cell death, and inhibited Ca^2+^ release induced by taxol (P<0.05). Detections in Twist1 silencing cells also confirmed this result. This study provided evidence that alterations of Twist1 expression modulates the chemosensitivity of FaDu cells to taxol. Therefore, *Twist1* knockdown may be a promising treatment regimen for advanced hypopharyngeal carcinoma patients with MDR.

## Introduction

Chemotherapy today remains the central therapeutic approach for the majority of cancer patients, as a treatment that places emphasis on the quality of life and the preservation of organ function (1,2). However, the application of chemotherapy is limited due to numerous obstacles, including adverse effects and multidrug resistance (MDR), which ultimately reduces treatment efficacy and are associated with disease progression (3,4).

MDR is one of the main causes of treatment failure and high mortality rates in disease, for patients with inherent resistance to drugs and for those who acquire resistance during treatment (5–7). In cancer, MDR is a multifactorial process, defined as a simultaneous resistance to several different types of commonly used antineoplastic agents, which has a severe impact on the therapeutic effect of these treatments (8). Therefore, clarifying the mechanisms and investigating the key elements regulating MDR, is critical for improving chemotherapy efficacy in the treatment of cancer. Extensive studies investigating drug resistance and resistance chemosensitivities have identified numerous genes and proteins associated with MDR. For example, certain membrane proteins are important in the development of MDR, notably MDR1 (MDR1/P-gp), MDR protein (MRP) and breast cancer resistance protein (BCRP), which is a member of the ATP binding cassette (ABC) transporter family that encode efflux pumps (9–12). Overexpression of these transporters has been reported to be correlated with the chemosensitivities of numerous chemotherapeutic agents (13). Nevertheless, MDR has not been prevented or effectively controlled in clinical practice. Further exploration for new regulators of MDR is urgently required, to improve the efficacy and application of chemotherapy as a cancer treatment.

*Twist1* is a highly conserved transcription factor which belongs to a basic helix-loop-helix family. Previous studies confirmed that overexpression of Twist1 was identified in multiple types of cancer in humans, with numerous damaging consequences, including promoting the immigration and invasion of cancer cells, and decreasing sensitivity to chemotherapy (14–16). Overexpression of Twist1 may be key to tumor drug resistance, but the precise mechanisms underlying this effect remain elusive. At present, no studies have investigated the role of Twist1 in taxol-exerted MDR on FaDu cells, or reported the possible role of Twist1 on FaDu cell apoptosis sensitivity. In an attempt to ascertain the role of Twist1 during MDR and clarify its mechanism of apoptosis sensitivity, a MDR cell line of FaDu cells was established and the stable transfections targeted to Twist1 overexpression and Twist1 silenced expression in FaDu cells were conducted. Chemosensitivity was studied in MDR cells and cells with variable expression levels of Twist1.

## Materials and methods

### Cells and reagents

The human hypopharyngeal carcinoma cell line FaDu was obtained from the American Type Culture Collection (ATCC; Manassas, VA, USA). Media and serum were purchased from Gibco (Invitrogen Life Technologies, Carlsbad, CA, USA). Anti-Twist1, activated caspase-3, activated caspase-9, Bcl-2, Bax and β-actin antibodies were purchased from Santa Cruz Biotechnology (Santa Cruz, CA, USA). The BCA protein assay kit was a product of Shenergy Biocolor Bioscience & Technology Company (Shanghai, China). The RevertAid First Strand cDNA Synthesis kit was obtained from Fermentas (Burlington, Ontario, Canada). All reagents were purchased from Sigma (St. Louis, MO, USA).

### Cell culture

FaDu/T was developed and determined as previously described (17). FaDu and FaDu/T cells were cultured as a monolayer on Dulbecco’s Modified Eagle’s Medium (DMEM; Gibco) containing 10% fetal calf serum, 100 U/ml penicillin and 100 mg streptomycin at 37°C in a humidified atmosphere composed of 95% air and 5% CO_2_.

### Assessment of cell viability and IC_50_ determination

Cells (5×10^4^/ml) sub-cultured in a 96-well cell culture cluster (Corning, Tewksbury, MA, USA) were treated with different concentrations of taxol. MTT (5 mg/ml, 20 μl) was added to each well 4 h prior to the indicated time points. Following 4 h of incubation at 37°C, the medium was removed and the precipitate was dissolved in dimethylsulfoxide. Then, the optical density (OD) values were measured at 570 nm using an ELISA reader (Multiskan MK3, Shanghai Bio-excellent, Shanghai, China). Relative cell viability was calculated according to the following formula: Cell relative viability (%) = OD^experiment^/OD^control^ × 100% (OD blank was used to zero). The IC_50_ was defined as the drug concentration required to decrease the cell viability to 50% of the control (no drug) value.

### Morphological observation for the apoptosis of cells

FaDu, FaDu/T and FaDu cells treated with taxol (200 nM) for 24 h were seeded (15×10^4^/well) in 24-well dishes containing 1 ml culture medium to observe the morphological changes. Acridine orange (AO) staining and Hoechst/PI double staining were conducted as previously described (17).

### Plasmid constructions of pcDNA3.1-Twist1 and generation of microRNA-Twist1

Entire coding cDNA fragments of *Twist1* were amplified by RT-PCR and sub-cloned into the multi-cloning site of pcDNA3.1 vector (pcDNA3.1-Twist1). The primers for human full length *Twist1* amplification were as follows: Forward (F), 5′-CGAAGCTTGAGAGATGATGCAGGACGTGTC-3′; rev erse (R), 5′-GGAATTCCTAGTGGGACGCGGACATG-3′.

### Confirmation of final constructs by DNA sequencing

MicroRNA-Twist1 was generated using the Block-iT™ PolII miR RNAi expression vector kit with EmGFP (Invitrogen Life Technologies) according to the manufacturer’s instructions. Four pairs of oligo sequences targeted to Twist1 silencing were tested and a scrambled microRNA was used as a control. The sequences targeting the *Twist1* gene-coding region were annealed and inserted into the pcDNA6.2-GW/EmGFPmiR vector to generate the microRNA interfering expression vector. We selected one pair of the sequence in which Twist1 was silenced most effectively and constructed the stable transfectant with this sequence. Briefly, the most effective sequence of microRNA-Twist1 and scrambled control was as follows: Twist1-oligo-F, 5′-TGCTGCTGCCGGTCTGGCTCTTCCTCGTTTTGGCCACTGACTGACGAGGAAGACAGACCGG CAG-3′, Twist1-oligo-R, 5′-CCTGCTGCCGTCTGTCTTCCTCGTCAGTC AGTGGCCAAAACGAGGAAGAGCCAGACCGGCAGC-3′; control-F: 5′-tgctgAAATGTACTGCGCGTGGAGACGTTTTGGCCACTGACTGACGTCTCCACGCAGTACATTT-3′, control-R: 5′-cctgAAATGTACTGCGTGGAGACGTCAGTCAGTGGCCAAAACGTCTCCACGCGCAGTACATTTc-3′.

### Generation of stable transfectants

Cell transfection was conducted using Lipofectamine 2000 (Invitrogen Life Technologies) according to the manufacturer’s instructions. Briefly, cells were grown to 80–90% confluence without antibiotics. Vectors containing the different constructs (10 μg) were diluted in opti-MEM (250 μl) and then mixed with the transfection solution for 20 min at room temperature. Following washing, cells were incubated with the transfection mixture at 37°C for 6–8 h and then were allowed to grow in fresh media.

Stable transfectant with pcDNA3.1/Twist1, named as ‘FaDu/Twist1+’, was isolated by selection with 500 mg/ml of G418 (Amresco, Solon, OH, USA) for 2~4 weeks. Cells with pcDNA3.1 vector were established as a negative control at the same time.

Stable transfectant with microRNA-Twist1, named as ‘FaDu/Twist1-miRNA’, was isolated by selection with blasticidin (2.5 μg/ml) and GFP under blue excitation. At the same time, cells with the above scrambled microRNA were built as a control as described above.

### RNA extraction and RT-PCR

Total RNA was extracted using TRIzol (Invitrogen Life Technologies). Expression of Twist1 mRNA was determined by RT-PCR with M-MuL V reverse transcription (Takara Bio, Inc., Shiga, Japan). All operations were conducted under the guidance of the manufacturer’s instructions. The primers were as follows: Twist1-F, 5′-GGAGTCCGCAGTCTTACGAG-3′; Twist1-R, 5′-TCTGGAGGACCTGGTACAGG-3′; β-actin-F, 5′-CTCCTTAATGTCACGCACGATTT-3′; β-actin-R, 5′-GTGGGGCGCCCCAGGCACCA-3′.

### Protein extraction and western blot analysis

Protein extraction and western blotting were conducted as previously described (17). Bands for β-actin, Twist1, cleaved caspase-3, cleaved caspase-9, Bcl-2 and Bax were visualized at apparent molecular weights of 43, 170, 28, 17, 37, 26 and 23 kDa, respectively. The relative OD ratio was calculated with Image J software (National Institutes of Health, Bethesda, MD, USA) by comparison to β-actin from three independent experiments.

### Intracellular calcium measurements

Cells with variable expression of Twist1 (FaDu, FaDu/T-200 nM, FaDu/Twist1+ and FaDu/Twist1-miRNA) were sub-cultured (5×10^4^/ml) in 6-well cell culture clusters (Corning). Following overnight growth, cells were treated with taxol (200 nM) for 24 h. Ca^2+^ concentrations were examined using Flou-3/AM (5 μM, 37°C for 30 min). Equal PBS was used as a control. At the end of the incubation, cells were examined under flow cytometry. The experiment was repeated three times.

### Statistical analysis

Data are presented as the mean ± standard error of the mean (SEM). Statistical calculations were performed using SPSS 16.0 software package (SPSS, Inc., Chicago, IL, USA). One-way analysis of variance (ANOVA) was applied to analyze the comparison of the means greater than or equal to three groups. P<0.05 was considered to indicate a statistically significant difference.

## Results

### Twist1 and MDR1/P-gp levels increase in a MDR-dependent manner in FaDu/T cells

**Figure 1:**
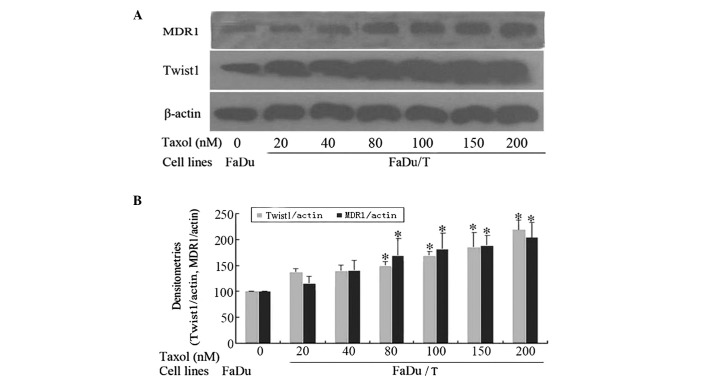
Twist1 and MDR1 were elevated in a MDR dose-dependent manner. (A) Twist1 and MDR1 were elevated in FaDu/T cells compared with FaDu cells. In high-fold resistant cells, Twist1 and MDR1 were elevated higher, which suggested that Twist1 and MDR1 changed in a MDR dose-dependent manner during MDR. (B) Image J software analysis shows that the significance of MDR1 change can be detected from FaDu/T-80 nM cells (^*^P<0.05 vs. FaDu cells). MDR, multidrug resistance.

Compared with FaDu cells, FaDu/T cells expressed higher levels of Twist1 and MDR1/P-gp in a MDR-dependent manner. A significant difference was identified from the FaDu/T cells, whose endurance to taxol was 80 nM (Fig. 1).

### Apoptosis sensitivity in FaDu/T cells

**Figure 2:**
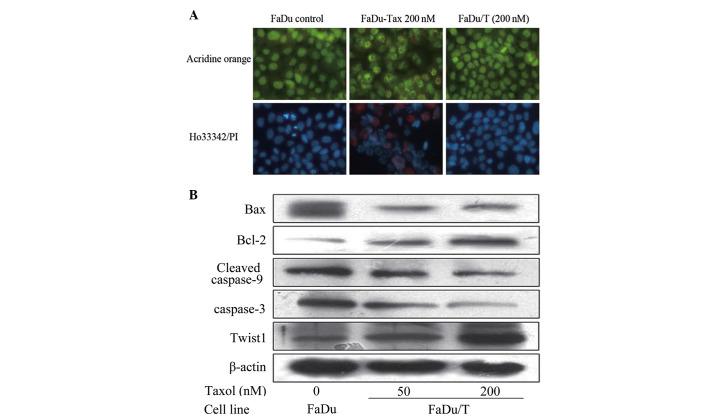
Morphological observation and detection of apoptosis-related proteins by western blot analysis. (A) AO staining and Ho.33342/PI double staining exhibited the anti-apoptosis of FaDu/T cells. (B) Cleaved caspase-3, caspase-9, Bcl-2 and Bax all changed to resist apoptosis during MDR progress. MDR, multidrug resistance; AO, acridine orange.

As illustrated in Fig. 2A, by AO staining, FaDu cells had a polygonal shape, but cells treated with taxol (200 nM) for 24 h became rounded and exhibited cytoplasmic contraction and chromatin condensation. Apoptotic bodies, the main morphological characteristic of apoptosis, were also present. However, FaDu/T cells had a similar morphology to FaDu cells, with intact polygonal nuclei.

For Ho.33342/PI double staining, blue intact nuclei can be observed, as in FaDu cells, red staining was interpreted as necrosis, while blue nuclear fragmentation was an indication of apoptosis. Compared with FaDu cells, red nuclei and blue nuclear fragmentation could be detected in cells treated with taxol (200 nM) for 24 h. However, FaDu/T cells (the endurability to taxol was 200 nM) demonstrated a similar morphology to FaDu cells, with blue intact nuclei. Our data indicated that FaDu/T cells exhibited anti-apoptosis activity when stimulated by taxol (Fig. 2A).

### Changes in apoptosis-related proteins in FaDu/T cells

Compared with FaDu cells, FaDu/T cells demonstrated apoptotic resistance, with cleaved caspase-3, cleaved caspase-9, Bcl-2 and Bax, all altered to resist apoptosis (Fig. 2B).

### Generation and determination of stable transfectants of Twist1 in FaDu Cells

**Figure 3:**
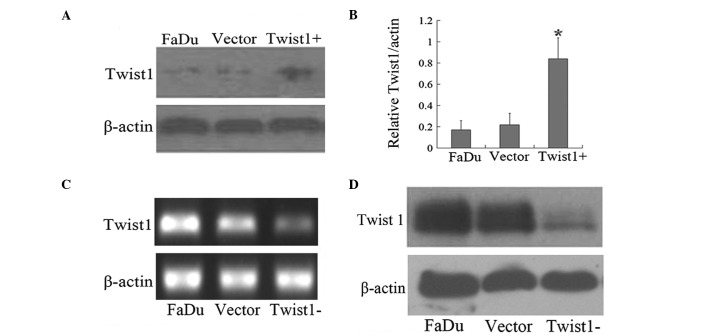
Establishment and determination of stable cell lines expressing Twist1 cDNA and Twist1 miRNA of FaDu cells. (A) Western blot analysis determination of Twist1 expression level. (B) Semi-quantitative assay of Twist1 based on Image J software analysis. (C) mRNA level of Twist1 in FaDu, negative control cells (Vector) and FaDu-Twist1-miRNA cells (Twist1−). (D) Protein level of Twist1 in FaDu, negative control cells (Vector) and FaDu-Twist1-miRNA cells (Twist1−).

**Figure 4:**
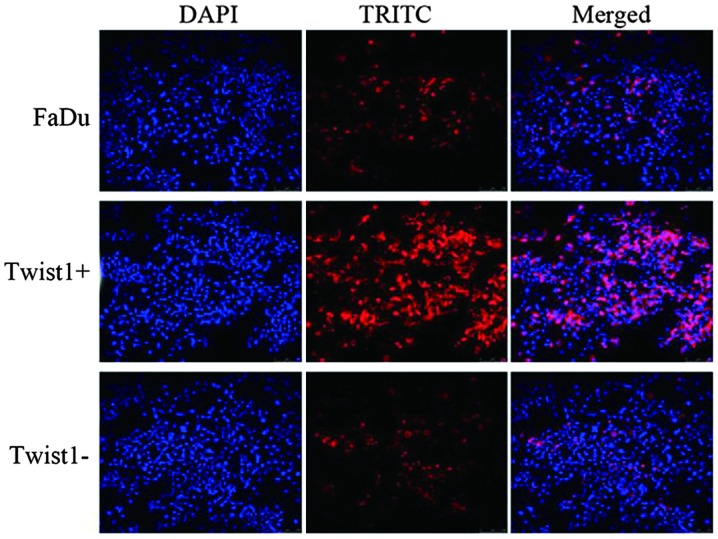
Twist1 expression detection by laser scanning confocal microscopy. DAPI was used to mark nuclei and TRITC was employed to indicate Twist1. Under the same intensity, FaDu-Twist1+ cells (Twist1+) expressed more Twist1 protein compared with FaDu cells. Reversely, FaDu-Twist1-miRNA cells (Twist1−) expressed less.

As summarized in Fig. 3, FaDu/Twist1+ cells exhibited a higher Twist1 expression level compared with FaDu cells and negative control cells (Fig. 3A and B; P<0.05). mRNA and protein levels of Twist1 in FaDu/Twist1-miRNA cells were detected, compared with the negative control cells and FaDu cells (Fig. 3C and D). Laser scanning confocal microscopy detected a consistent expression of Twist1 with above in FaDu cells, FaDu/Twist1+ cells and FaDu/Twist1-miRNA cells, respectively (Fig. 4).

### Twist1 regulates the chemosensitivity of FaDu cells to taxol

**Figure 5:**
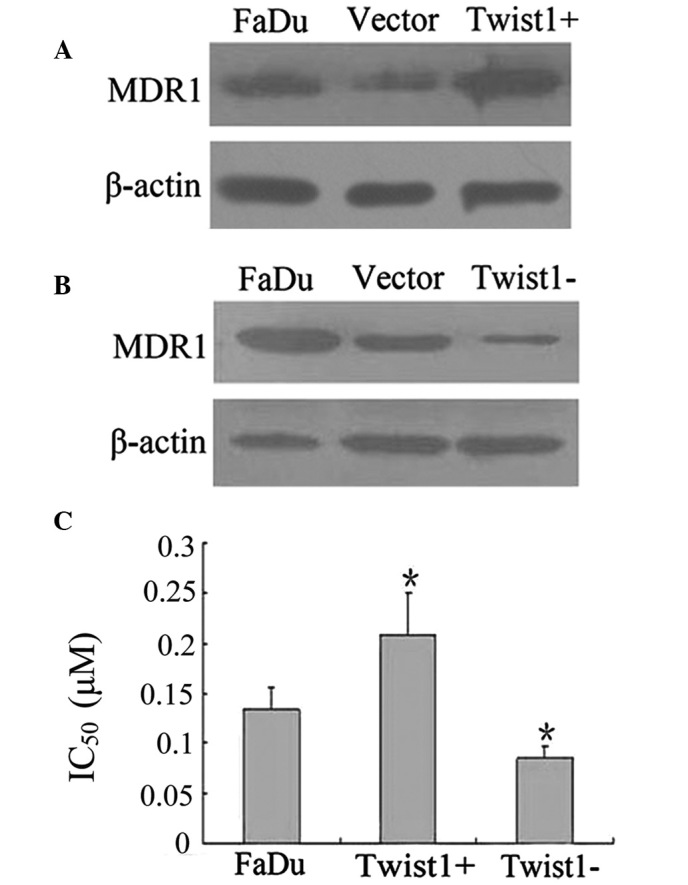
Chemosensitivity and MDR1 changes with expression of Twist1. (A and B) MDR1 increased in FaDu-Twist1+ cells (Twist1+) and decreased in FaDu-Twist1-miRNA cells (Twist1−), compared with FaDu and negative control cells (Vector). (C) IC_50_ was applied to chemo-drug sensitivity of cells with different Twist1 expressions.^*^P<0.05 vs. FaDu cells. MDR, multidrug resistance.

MDR1/P-gp was elevated or downregulated accompanied by corresponding changes in Twist1, which demonstrated that Twist1 may positively regulate MDR1/P-gp expression levels (Fig. 5A and B). The IC_50_ was further analyzed in the transfectants. As illustrated in Fig. 5C, IC_50_ of FaDu/Twist1+ cells was 0.208±0.042 μM, and for FaDu-Twist1-miRNA cells was 0.085±0.012 μM, which were significantly different compared with FaDu cells (IC_50_=0.134±0.022 μM; P<0.05). The data suggests that overexpression of Twist1 protected the cell from taxol damage and decreased the chemosensitivity of FaDu.

### Changes in apoptosis-related proteins and Ca^2+^ release

**Figure 6:**
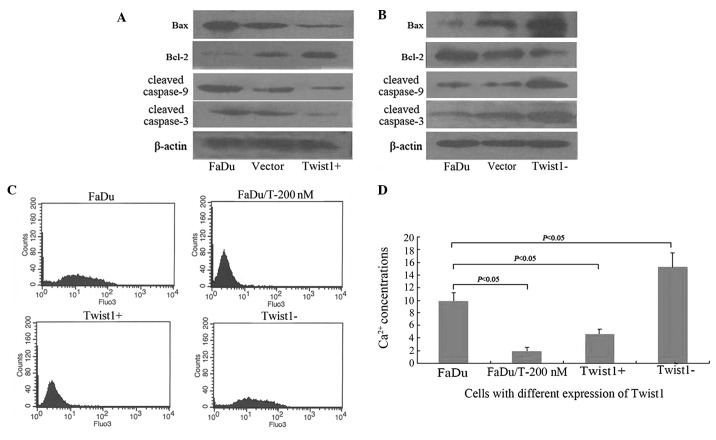
Apoptosis-related proteins assayed by western blot analysis and Ca^2+^ concentrations analyzed by flow cytometry with expression of Twist1. (A and B) Cleaved caspase-3, caspase-9, Bcl-2 and Bax changed accordingly to resist apoptosis in FaDu/Twist1+ cells (Twist1+), and changed reversely in FaDu/Twist1-miRNA cells (Twist1−) to accelerate apoptosis, compared with FaDu, FaDu cells with negative control vector (Vector). (C and D) Ca^2+^ concentrations analyzed in different cell lines. A significant difference could be found with the expression of Twist1 (P<0.05).

In FaDu/Twist1+ cells, cleaved caspase-3, cleaved caspase-9, Bcl-2 and Bax, all demonstrated alterations with the purpose of resisting apoptosis (Fig. 6A). By contrast, in FaDu/Twist1-miRNA cells, cleaved caspase-3, cleaved caspase-9, Bcl-2 and Bax all changed accordingly, with the purpose of promoting apoptosis (Fig. 6B). Results proved that Twist1 regulated the apoptosis sensitivity of FaDu cells. Overexpression of Twist1 decreased the sensitivity of apoptosis in FaDu cells.

### Twist1 overexpression inhibits Ca^2+^ release induced by taxol

Mean counts of Ca^2+^ released in FaDu, FaDu/T-200 nM, FaDu/Twist1+ and FaDu/Twist1-miRNA cells were 9.89±1.35, 1.96±0.57, 4.7±0.66 and 15.22±2.24, respectively (Fig. 6C). As summarized in Fig. 6D, statistical analysis revealed that the Ca^2+^ concentration was significantly elevated under taxol for 24 h in FaDu cells, which may be inhibited in FaDu/T-200 nM. By contrast, Ca^2+^ elevation induced by taxol could be buffered by Twist1 overexpression and significantly aggravated by Twist1 silencing (P<0.05). These results suggested that Ca^2+^ was reduced during MDR, and that Twist1 may regulate the sensitivity of cells to factors inducing Ca^2+^ release.

## Discussion

MDR is a multi-factorial process defined as the simultaneous resistance to several different types of commonly used antineoplastic agents. The main mechanism of MDR involves the exclusion of drugs from the cell by overexpression of either MDR1/P-glycoprotein (MDR1/P-gp; a type of glycoprotein responsible for drug exclusion) or various members of the MRP family (8). A number of other factors are considered to be involved, including the alteration of levels or properties of drug targets, increasing detoxification due to enhanced activity of glutathione S-transferase, preventing activation of drug to its active form and enhancing repair capability of the cell following injury (18,19). Although extensive studies have investigated MDR, the effect observed in clinical practice is of little significance. MDR remains as the central reason for chemotherapy failure in cancer therapeutics. Therefore, further exploration for the development of a new regulator of MDR is urgently required.

*Twist1* is a highly conserved transcription factor and is a member of a basic helix-loop-helix family. In our previous study, we reported that Twist1 may be critically involved in taxol-induced apoptosis of Hep-2 cells (20). Several other studies have also been exploring the associations of chemosensitivity and Twist1, in an attempt to further our understanding of the mechanisms of MDR (21,22). Zhuo *et al* raised the possibility of Twist1 depletion as a promising approach to lung cancer therapy, in a short interfering RNA study directed against Twist1 on A549 (23). However, studies identifying the function of Twist1 in the chemosensitivity of hypopharyngeal carcinomas are lacking. Our recent findings demonstrated that the MDR cell line of Hep-2, induced by taxol, was more invasive than its parent cell line, which was to a certain extent, mediated through the overexpression of MDR1/P-gp/P-pg (24). In the present study, the upregulation of MDR1/P-gp and Twist1 in MDR FaDu/T cells was detected, which suggest the expression levels of MDR1/P-gp and Twist1 were positively correlated with MDR progression in hypopharyngeal carcinomas. On the assumption that certain correlations may exist between MDR1/P-gp and Twist1, DNA recombinant and cell transfection experiments were conducted. Stable transfections characterized by overexpression of Twist1 and Twist1 silencing were established. It was identified that Twist1 overexpression led to the upregulation of MDR1/P-gp, and reversely, Twist1 silencing led to the decrease of MDR1/P-gp. The results reliably proved that Twist1 modulated the MDR1/P-gp expression level. These findings are consistent with several other studies identifying similar effects in other cell types. Overexpression of Twist1, Snail and FOXC2 are considered to increase the promoter activity of ABC transporters (25).

As the main regulator of MDR, manipulation of MDR1/P-gp expression may effect chemosensitivity. Specific functions must be ultimately implemented. Therefore, the chemosensitivities of stable transfectants with variable levels of Twist1, were detected by MTT. IC_50_ values were used to assess chemosensitivities and the results proved our hypothesis was appropriate. The IC_50_ values in cells with overexpression of Twist1 was markedly increased, meaning that Twist1 upregulation decreased the chemosensitivity of cells and Twist1 silencing reversely increased it. Similar findings in advanced and/or metastatic bladder (BCa) and prostate (PCa) cancer types support these findings (26). They identified that Twist1 may be utilized as a molecular target to restore chemosensitivity in BCa and prostate PCa cancer types.

In hypothesizing how Twist1 modulates chemosensitivities, it may be possible that numerous changes occur with Twist1 expression, such as alteration of drug metabolism, derangement of intracellular pathway signaling, cross-talk between different membrane receptors and modification of apoptotic signaling. In the present study, the focus was on the modification of apoptosis (20). It has been confirmed that proteins of the Bcl-2 family, which generally repress apoptosis (Bcl-2 and Bcl-xL) or promote apoptosis (Bax, Bak and Bad), are important in regulating the activation of caspases (27–29). These proteins, to a certain degree, affect caspase activation by controlling the release of cytochrome C from the mitochondria, which in turn interacts with the adapter protein Apaf-1, resulting in the activation of pro-Caspase-9 (30). The disturbance of intracellular free Ca^2+^ ions is another key event when cells are exposed to damaging stress (31–33). In the present study, apoptosis was inhibited in MDR cells. Bcl-2 expression was upregulated, while expression of Bax, cleaved caspase-3 and caspase-9 were decreased. Detection of stable transfectants demonstrated that apoptosis sensitivity decreased in cells which overexpressed Twist1, with Bcl-2 increased and Bax cleaved caspase-3 and caspase-9 all downregulated. Furthermore, after Twist1 was silenced by targeted micro-RNA, the apoptosis sensitivity of these cells increased, with all the above apoptotic-related proteins altered to sensitize apoptosis. Also, it was identified that taxol led to an increase in intracellular free cytosolic Ca^2+^, which may be partially attenuated by Twist1 overexpression and aggravated by Twist1 silencing. These data strongly indicated that Twist1 expression may regulate apoptosis sensitivity, Bcl-2 and caspase family proteins (which were involved in Twist1-mediated processes) and taxol-triggered apoptosis in FaDu cells, at least in part, in the participation of intracellular Ca^2+^. Studies on pancreatic cancer also concluded a similar phenomenon (34). Evidence in the nasopharyngeal carcinoma cell line HNE1 revealed a downregulation of Twist1 may increase drug sensitivity of HNE1 to taxol by inducing apoptosis (35). To the best of our knowledge, this is the first study to report the function of Twist1 in apoptosis sensitivity of hypopharyngeal cancer, yet the report about the regulation of Twist1 to Ca^2+^ influx has not appeared. It was identified that the Ca^2+^ influx caused by taxol may be attenuated by Twist1 overexpression. This may provide novel molecular mechanisms of targeted gene therapy of head and neck squamous cell cancer to Twist 1, and combining the sensitizer of Ca^2+^ infux may enhance the chemosensitivity of Twist1-targeted chemotherapy.
